# Supplementary material for: Trafficking regulator of GLUT4-1 (TRARG1) is a GSK3 substrate
Source: Biochem J. 2022 Jun 13;479(11):1237–56. doi: 10.1042/BCJ20220153 (PMC9284383; doi:10.1042/BCJ20220153)
Supplement: Supplementary Material [file BCJ-479-1237-s1.pdf]

Supplemental Figure S1, relevant to Figure 3. (A) Subcutaneous white adipose tissue (SWAT) was excised from mice and minced. Explants were serum-starved in DMEM/2% BSA/20 mM HEPES, pH 7.4 for 2 h followed by treatment with insulin (10 nM) or LY2090314 (GSK3i) (500 nM) for 30 min at 37 ^{o}C. Treatment was terminated and tissues were solubilized in RIPA buffer and subjected to analysis by immunoblotting. Apparent higher molecular weight TRARG1 bands in subcutaneous white adipose tissue (SWAT) explants were reduced in intensity by insulin or GSK3 inhibitor treatment. (B) Quantification of (A). The ratio of apparent higher molecular weight (HMW) TRARG1 signal to total TRARG1 signal was quantified (n=4, mean±SEM, **p* <0.05; ***p* <0.01; ****p* <0.001, comparisons with basal condition). (C) Apparent higher molecular weight TRARG1 bands in human SGBS adipocytes were reduced in intensity by insulin or GSK3 inhibitor treatment. Number below blot are the HMW/total TRARG1 ratio. (D-F) Treatment of L6 myotubes (D) (Empty vector control; EV), HEK-293E cells (E), HeLa cells (F) or with GSK3 inhibitors reduced the intensity of apparent higher molecular weight TRARG1 bands. In L6 myotubes, 20 min 100 nM insulin treatment also reduced the intensity of the apparent higher molecular weight TRARG1 bands. For panels A, C, D, E and F, the migration positions of molecular mass markers (kilodaltons) are shown to the right. Higher molecular weight (HMW) TRARG1 bands indicated by arrow.

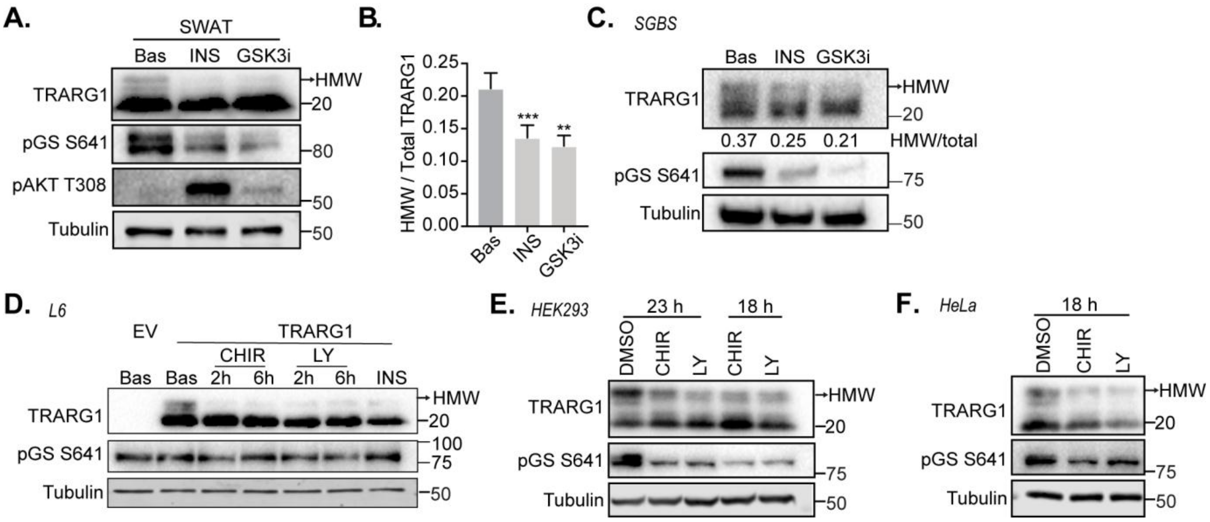

Supplementary table S1. Identified post-translational modifications on murine Trarg1.

Supplementary table S2. Analysis of *TRARG1* conservation and polymorphisms in placental mammals.
